# Supplementary material for: Glucoselipid Biosurfactant Biosynthesis Operon of Rouxiella badensis DSM 100043T: Screening, Identification, and Heterologous Expression in Escherichia coli
Source: Microorganisms. 2025 Jul 15;13(7):1664. doi: 10.3390/microorganisms13071664 (PMC12299268; doi:10.3390/microorganisms13071664)
Supplement: Supplementary file 1 [file microorganisms-13-01664-s001.zip › microorganisms-3733643-supplementary.pdf]

Supplementary Information

Glucoselipid Biosurfactant Biosynthesis Operon of *Rouxiella badensis* DSM 100043^T: Screening, Identification, and Heterologous Expression in *Escherichia coli*

Andre Fahriz Perdana Harahap[1], Chantal Treinen[1,2], Leonardo Joaquim Van Zyl[3], Wesley Trevor Williams[3], Jürgen Conrad[4], Jens Pfannstiel[5], Iris Klaiber[5], Jakob Grether[1], Eric Hiller[1], Maliheh Vahidinasab[1], Elvio Henrique Benatto Perino[1], Lars Lilge[1], Marla Trindade[3] & Rudolf Hausmann[1,*]

[1] Department of Bioprocess Engineering (150k), Institute of Food Science and Biotechnology, University of Hohenheim, Fruwirthstr. 12, Stuttgart 70599, Germany; andrefahrizperdana.harahap@uni-hohenheim.de (AFPH); jakob.grether@uni-hohenheim.de (JG); eric.hiller@uni-hohenheim.de (EH); malihe.vahidinasab@uni-hohenheim.de (MV); eperino@uni-hohenheim.de (EHBP); lars.lilge@uni-hohenheim.de (LL)

[2] Cellular Agriculture, TUM School of Life Sciences, Technical University of Munich, 85354 Freising, Germany; chantal.treinen@tum.de (CT)

[3] Department of Biotechnology, Institute for Microbial Biotechnology and Metagenomics (IMBM), University of the Western Cape, 7535 Cape Town, South Africa; wesleywt@gmail.com (WTW); vanzyllj@gmail.com (LJVZ); prof.marlatt@gmail.com (MT)

[4] Department of Organic Chemistry (130b), Institute of Chemistry, University of Hohenheim, Garbenstr. 30, Stuttgart 70599, Germany; juergen.conrad@uni-hohenheim.de (JC)

[5] Core Facility Hohenheim, Mass Spectrometry Unit, University of Hohenheim, Ottilie-Zeller-Weg 2, 70599 Stuttgart, Germany; jens.pfannstiel@uni-hohenheim.de (JP); iris.klaiber@uni-hohenheim.de (IK)

* Correspondence: rudolf.hausmann@uni-hohenheim.de (RH)

Table S1. Primers used in this study.

Primer	Sequence (5′ → 3′)	Details
pCCERI-FVD1	CGATGCACATGCTGTATGCC	Sequencing clone 1.8.H6
pCCERI-RVS1	CAGCGCAAAAACCTTCGTGT	Sequencing clone 1.8.H6
pCCERI-FVD2	TTCCGCCATTCCTATGCGAT	Sequencing clone 1.21.F4
pCCERI-RVS2	ACATCCAGCGCAAAAACCTTC	Sequencing clone 1.21.F4
pORF-FVD	CATATGTTACCTGAAGTTACTGTCAC	PCR of ORF positive clones
pORF-RVS1	CTCGAGTTTTTTTCTCCCTGCGCGATATTG	PCR of ORF1→2 of positive clones
pORF-RVS2	CTCGAGGTTTAGTTCGGCAACAGGCT	PCR of ORF1→3 of positive clones
pJET FVD	CGACTCACTATAGGGAGAGCGGC	colony PCR with the CloneJET PCR Cloning Kit®
pJET RVS	AAGAACATCGATTTTCCATGGCAG	colony PCR with the CloneJET PCR Cloning Kit®
S-1015	CTCAAGACCGTTTAGAGGC	Sequencing pCAT1 and pCAT2
S-1016	GTGGGATCCAAGCGTTATAGCG	Sequencing pCAT2
T7	TAATACGACTCACTATAGGG	Sequencing pCAT1 and pCAT2
pAFP1_F	CAAAATCGAAGATGACATCCATACAAGAA AGCCTTCAATTTATTCTGTTTTCG	Construction of pAFP1 by Gibson Assembly
pAFP1_R	GAAGTTGCTTTCTTGTATGGATGTCATCTTC GATTTTGACTTCACG	Construction of pAFP1 by Gibson Assembly
pAFP2_F	ACATCATCATTTTTCATATGTATATCTCCTT CTTAAAGTTAAAC	Construction of pAFP2 by Gibson Assembly
pAFP2_R	AAGAAGGAGATATACATATGAAAAATGAT GATGTGATCCTTC	Construction of pAFP2 by Gibson Assembly
pAFP3_F	AGTCAAAATCGAAGATGACATCCATATGT ATATCTCCTTCTTAAAGTTAAACAAAATTA TTTCTAGAGGG	Construction of pAFP3 by Gibson Assembly
pAFP3_R	AGAAATAATTTTGTTTAACTTTAAGAAGGA GATATACATATGGATGTCATCTTCGATTTTG ACTTCACG	Construction of pAFP3 by Gibson Assembly
pAFP5_F	CAGGGAGAAAAAAATAAGATCCGGCTGCT AACAAAGC	Construction of pAFP5 by Gibson Assembly
pAFP5_R	TTGTTAGCAGCCGGATCTTATTTTTTTCTCC CTGCGCGA	Construction of pAFP5 by Gibson Assembly

Table S2. ^{1}H and ^{13}C NMR chemical shifts, spin multiplicity, *J* coupling constants of compounds **1–3** of glucosemonolipid of fraction 37 (*E. coli* pAFP1) in methanol-*d*4 at 600 (^{1}H) and 150 MHz (^{13}C).

**Compound 1 (C-2 substituted Glu-C$_{10:0}$)**				
**Atom Number**	**α-Anomer**		**β-Anomer**	
	**δ$_H$ (ppm), multiplicity, *J* (Hz)**	**δ$_C$ (ppm)**	**δ$_H$ (ppm), multiplicity, *J* (Hz)**	**δ$_C$ (ppm)**
1	5.31, d, 3.6	91.23	4.66, d, 8.0	96.26
2	4.64, dd, 3.6, 9.8	75.55	4.72, dd, 8.0, 9.6	76.77
3	3.92, dd, 9.2, 9.8	72.17	3.55, t-like, 9.5[a]	76.19
4	3.44, t-like, 9.3	71.87	3.40, dd, 8.9, 9.3[a]	71.64
5	3.84, ov	72.97	3.35, ddd, 2.2, 5.5, 9.5[a]	76.81
6a/b	3.83, ov<br>3.73, dd, 5.8, 12.3	62.57	3.91, dd, 2.1, 11.7[a]<br>3.71, dd, 5.5, 12.0[a]	62.71
1′	-	173.14	-	172.70
2′	2.62, ov<br>2.53, ov	43.41	2,59, ov<br>2.53, ov	43.65
3′	4.07, ov	69.25[b]	4.07, ov	69.24[b]
4′	1.56, ov<br>1.52, ov	38.00	1.56, ov<br>1.52, ov	37.85
5′	1.51, ov<br>1.40, ov	26.69	1.51, ov<br>1.40, ov	26.66
6′	1.35, ov	30.65	1.35, ov	30.65
7′	1.34, ov	30.43	1.34, ov	30.43
8′	1.34, ov	33.02	1.34, ov	33.02
9′	1.36, ov	23.72	1.36, ov	23.72
10′	0.94, t, 7.1	14.43	0.94, t, 7.1	14.43
**Compound 2 (C-3 substituted Glu-C$_{10:0}$)**				
**Atom Number**	**α-Anomer**		**β-Anomer**	
	**δ$_H$ (ppm), multiplicity, *J* (Hz)**	**δ$_C$ (ppm)**	**δ$_H$ (ppm), multiplicity, *J*(Hz)**	**δ$_C$ (ppm)**
1	5.18, d, 3.6	93.89	4.61, d, 7.9	98.12
2	3.55, dd, 3.5, 10.0[a]	72.14	3.30, dd, 7.9, 9.8	74.52
3	5.28, t-like, 9.6	77.29	4.98, t-like, 9.6	76.19
4	3.53, t-like, 9.6[a]	69.82[c]	3.51, t-like, 9.4[a]	69.44[c]
5	3.90, ddd, 2.2, 4.7,10.1	72.83	3.41, ddd, 2.3, 5.7, 9.7[a]	77.80
6a/b	3.82, dd, 2.6, 11.9[a]<br>3.76, dd, 4.3, 11.5[a]	62.35	3.89, dd, 2.3, 12.1[a]<br>3.72, dd, 5.5, 11.9[a]	62.52
1′	-	173.63	-	173.38
2′	2.62, ov<br>2.53, ov	43.45	2,59, ov<br>2.53, ov	43.49
3′	4.07, ov	69.44[d]	4.07, ov	69.42[d]
4′	1.56, ov<br>1.52, ov	38.11[e]	1.56, ov<br>1.52, ov	38.02[e]
5′	1.51, ov<br>1.40, ov	26.73[f]	1.51, ov<br>1.40, ov	26.69[f]
6′	1.35, ov	30.65	1.35, ov	30.65
7′	1.34, ov	30.43	1.34, ov	30.43
8′	1.34, ov	33.02	1.34, ov	33.02
9′	1.36, ov	23.72	1.36, ov	23.72
10′	0.94, t, 7.1	14.43	0.94, t, 7.1	14.43
**Compound 3 (C-6 substituted Glu-C$_{10:0}$)**				
**Atom Number**	**α-Anomer**		**β-Anomer**	
	**δ$_H$ (ppm), multiplicity, *J* (Hz)**	**δ$_C$ (ppm)**	**δ$_H$ (ppm), multiplicity, *J*(Hz)**	**δ$_C$ (ppm)**
1	5.12, d, 3.5	94.01	4.61, d, 7.9	98.12
2	3.39, dd, 3.7, 9.5[a]	n.a,	3.30, dd, 7.9, 9.8	74.52
3	3.72, t-like, 9.3[a]	74.72	4.98, t-like, 9.6	76.19
4	3.37, dd, 9.2, 9.6[a]	n.a.	3.51, t-like, 9.4[a]	69.44[c]
5	4.01, ddd, 2.2, 4.8, 9.7[a]	70.61	3.41, ddd, 2.3, 5.7, 9.7[a]	77.80

6a/b	4.40, dd, 2.6, 11.8<br>4.27, dd, 5.2, 11.5[a]	64.95	3.89, dd, 2.3, 12.1[a]<br>3.72, dd, 5.5, 11.9[a]	62.52
1′	-	173.42[g]	-	173.44[g]
2′	2.62, ov<br>2.53, ov	43.56[g]	2,59, ov<br>2.53, ov	43.53[g]
3′	4.07, ov	69.36[g]	4.07, ov	69.32[g]
4′	1.56, ov<br>1.52, ov	38.22[g]	1.56, ov<br>1.52, ov	38.13[g]
5′	1.51, ov<br>1.40, ov	26.71[g]	1.51, ov<br>1.40, ov	26.71[g]
6′	1.35, ov	30.65[g]	1.35, ov	30.65[g]
7′	1.34, ov	30.43[g]	1.34, ov	30.43[g]
8′	1.34, ov	33.02[g]	1.34, ov	33.02[g]
9′	1.36, ov	23.72[g]	1.36, ov	23.72[g]
10′	0.94, t, 7.1	14.43[g]	0.94, t, 7.1	14.43[g]

ov: overlapped by other signals.

br: broad.

n.a.: not assigned; chemical shifts of minor component **3** could not unambiguously assigned due to overlap with the signals of major **2** and **3**.

[a] Coupling pattern and constants derived by selective 1D TOCSY.

[b, c, d, e, f,] maybe interchanged.

[g] ^{1}H and ^{13}C NMR signals completely overlapped and therefore tentatively assigned.

Coupling constants were directly taken from the spectra and are not averaged.

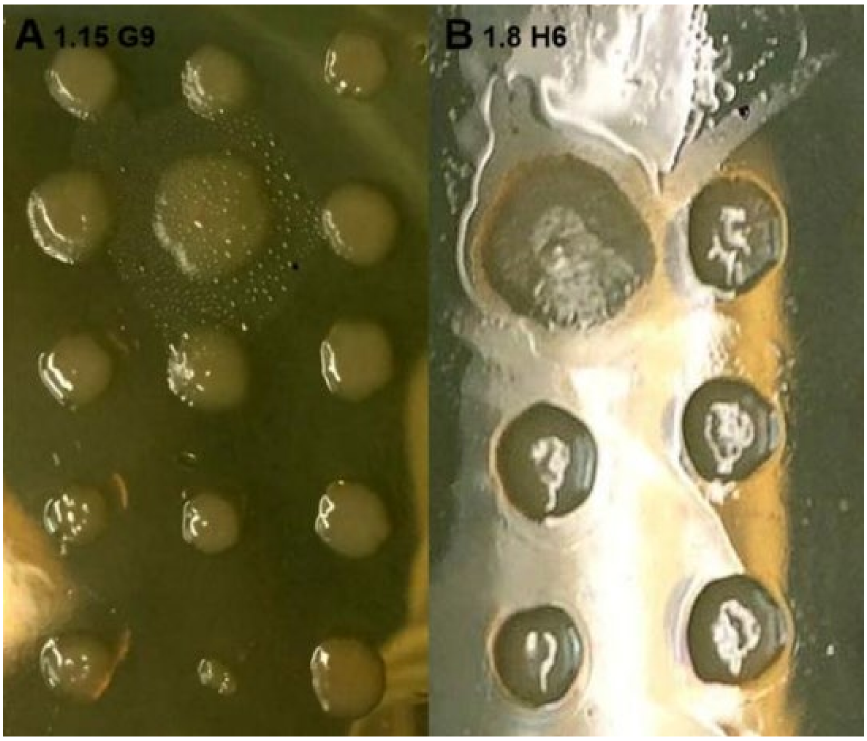

Figure S1. Halo formation of the clones 1.15 G9 and 1.8 H6 shown from four positive clones.

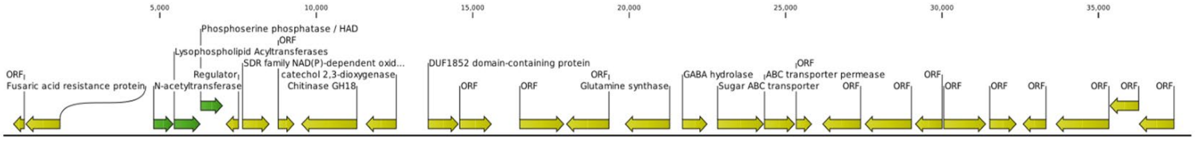

Figure S2. Open reading frames of sequenced *R. badensis* region. ORFs of interest are marked in green: N-acetyltransferase, lysophospholipid acyltransferase, and phosphoserine phosphatase/ HAD.

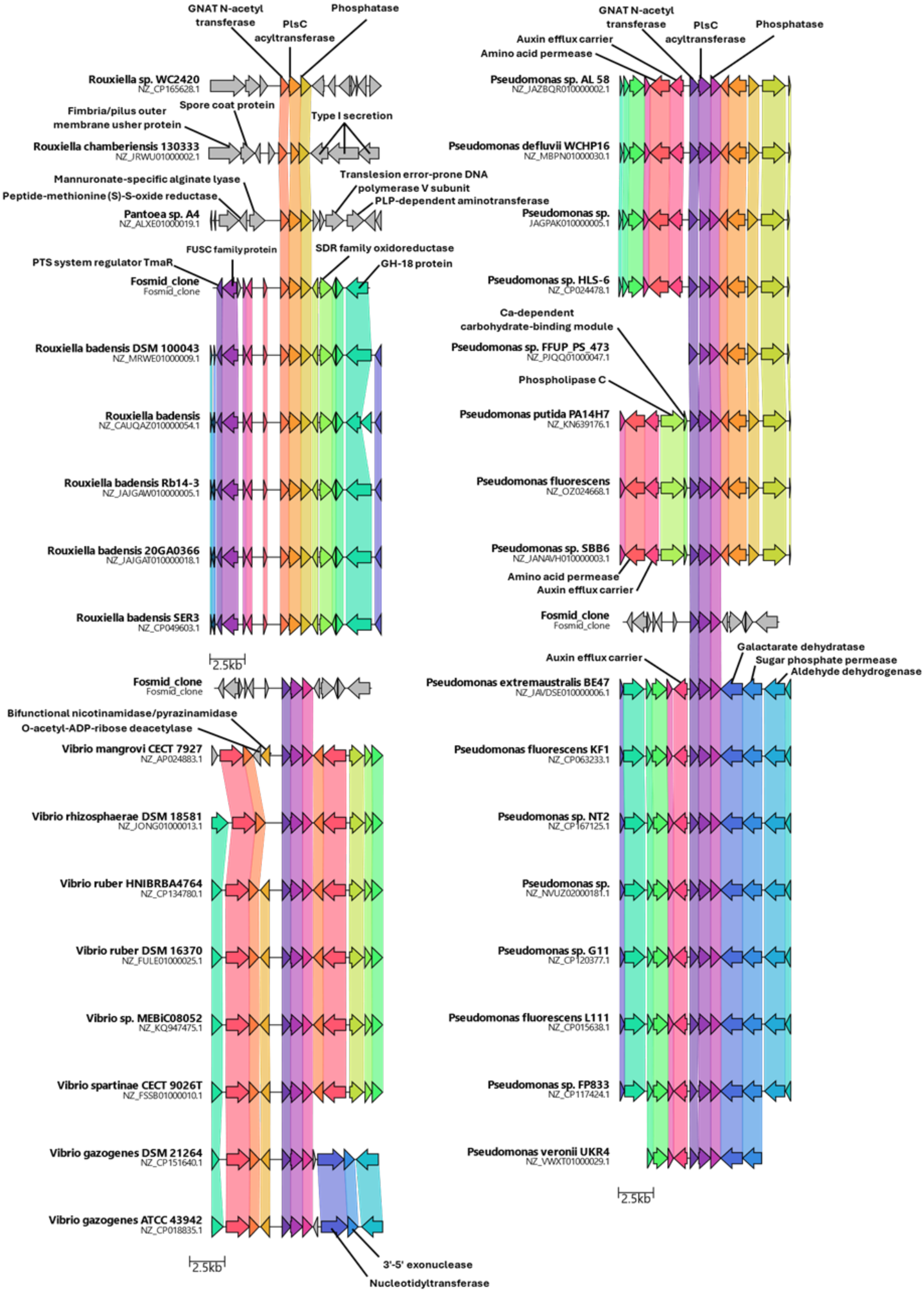

Figure S3. Extended comparison of genomic regions encoding glucoselipid pathway-related genes among different phyla.

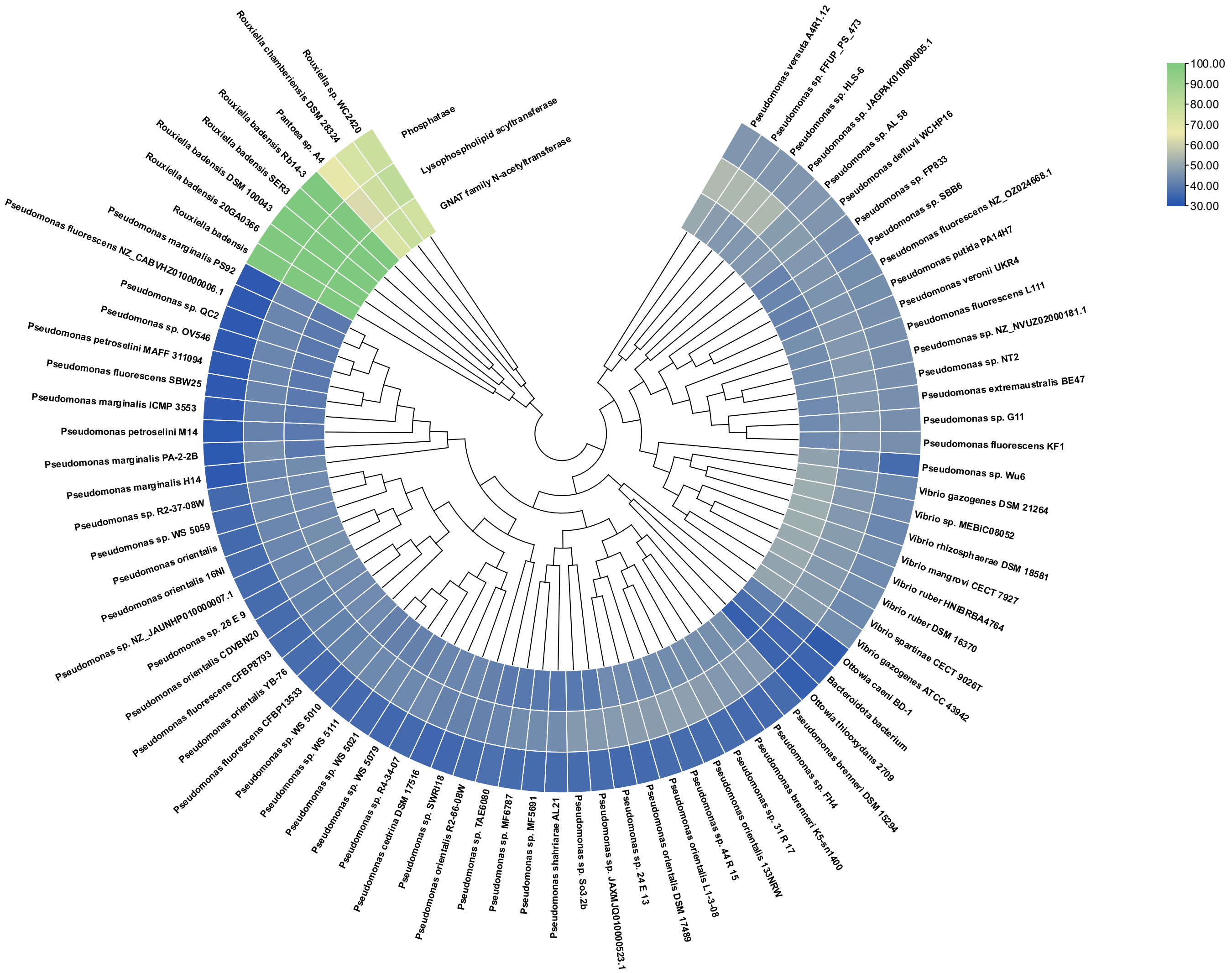

Figure S4. Comparison of amino acid similarity among glucoselipid synthesis pathway ORFs in bacteria that have retained all three ORFs. The cladogram represents hierarchical clustering where the similarities were calculated using Euclidean distance as the measure of dissimilarity. Clustering was performed using the complete linkage method.

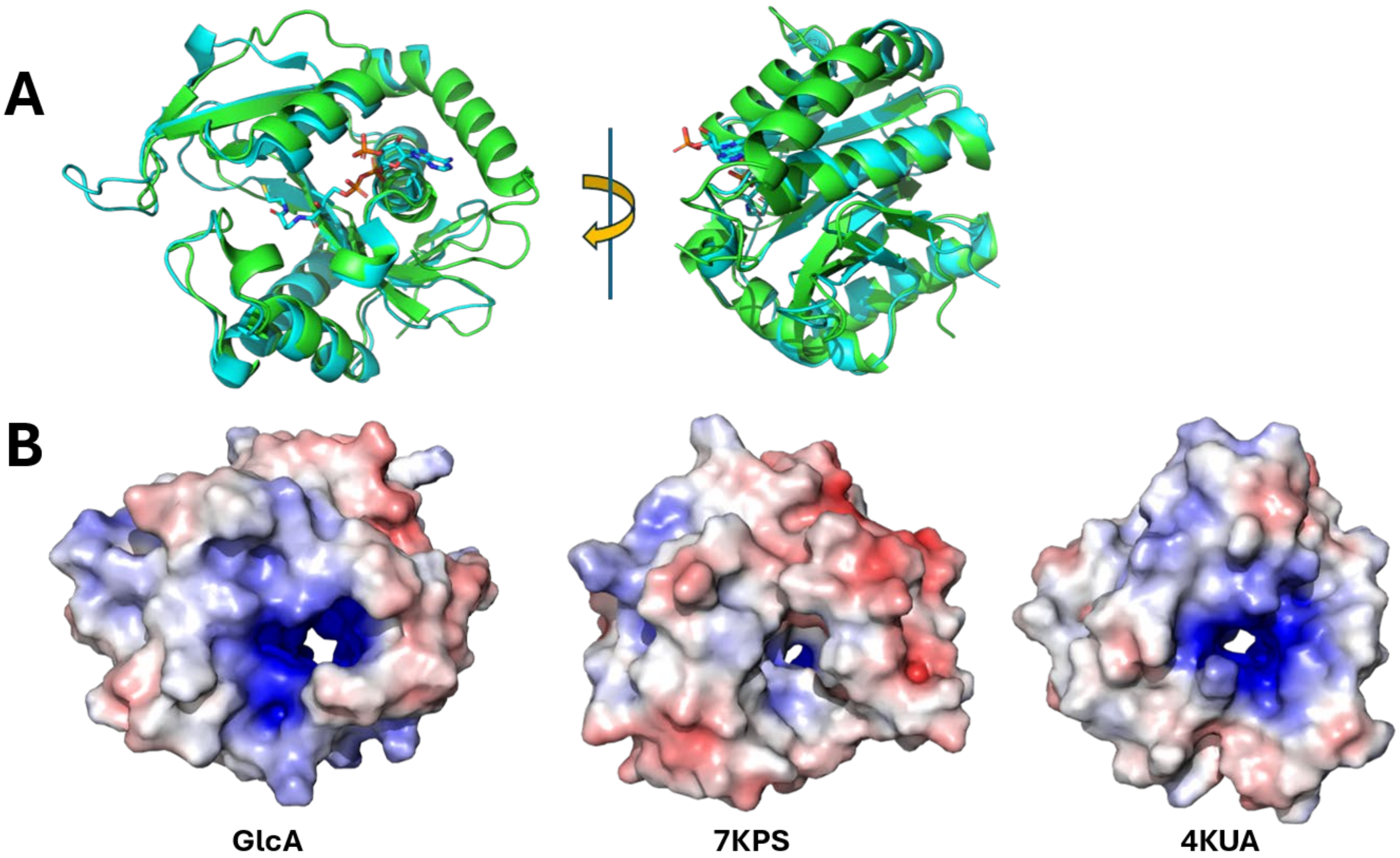

Figure S5. A) Cartoon representation of the structural alignment of GlcA (green) with 7KPS (cyan) with RMSD of 0.944. The structure of Coenzyme-A is shown as stick model in the binding pocket based on the 7KPS structure. B) Comparison of the surface geometry and charge ditribution on the acyl acceptor side of GlcA in comparison with 7KPS and 4KUA, known to catalyze O-acetylation of chloramphenicol.

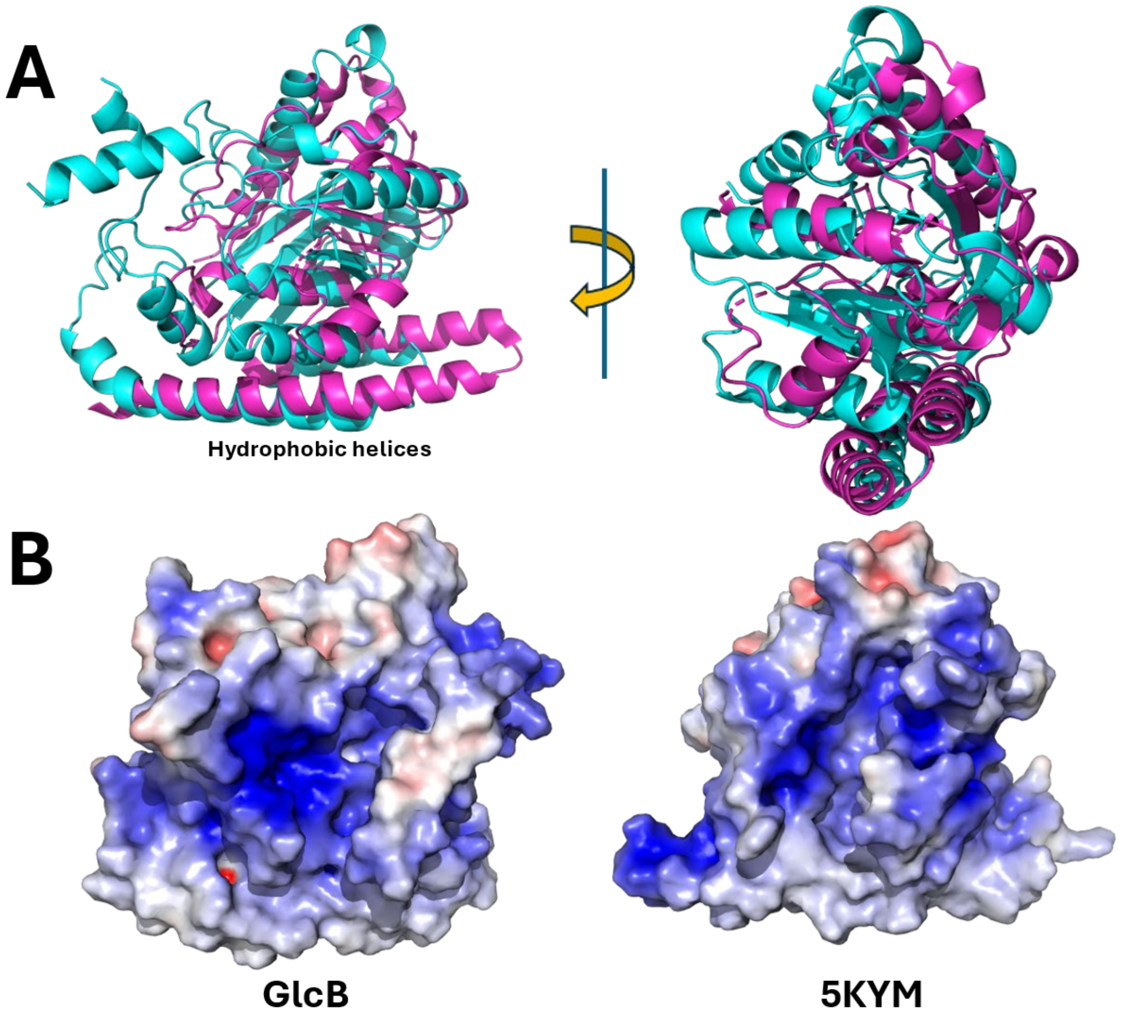

Figure S6. A) Cartoon representation of the structural alignment of GlcB (cyan) with 5KYM (purple) with RMSD of 4.7. B) Comparison of the surface geometry and charge ditribution of the catalytic cleft of GlcB in comparison with 5KYM known to catalyze acylation of 1 acyl-glycerol-3-phosphate.

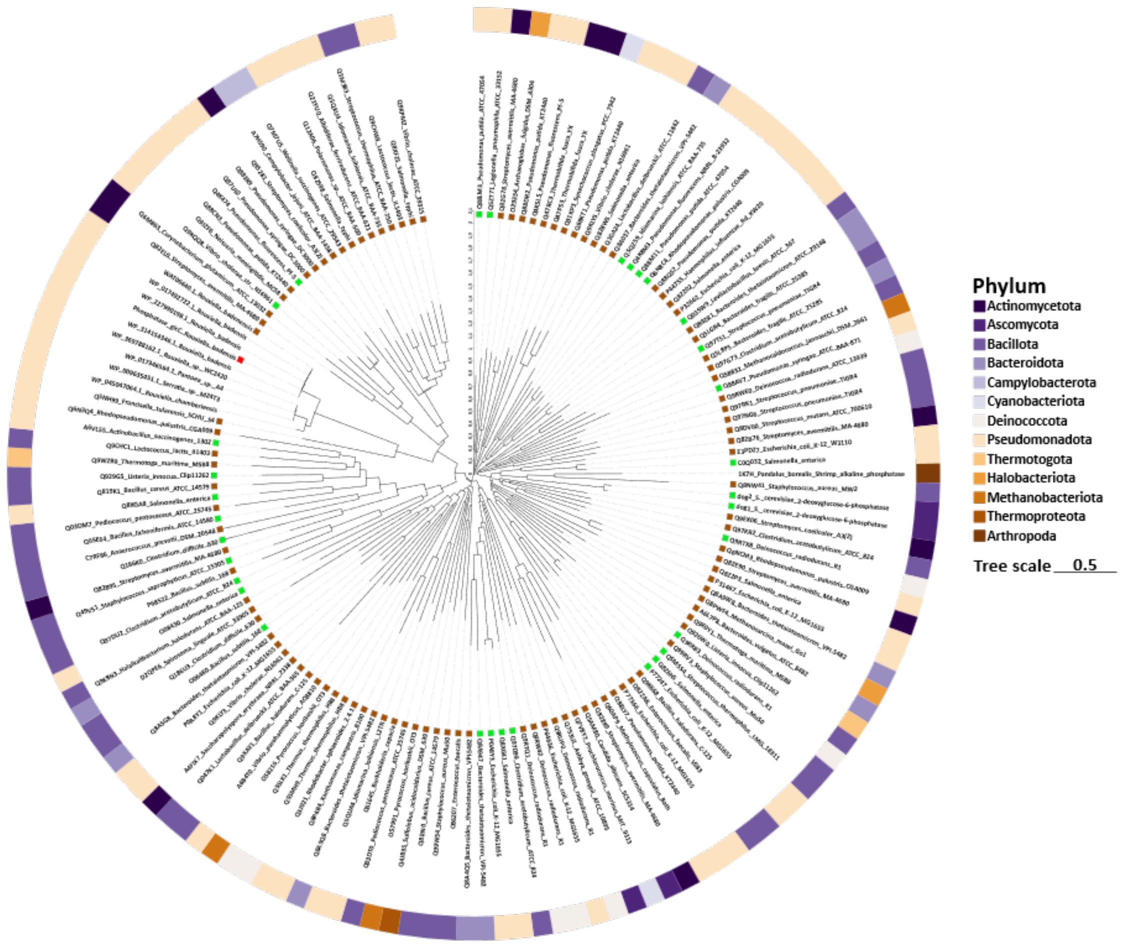

Figure S7. Structure-based phylogenetic relationship between all C1-phosphatase members that have been assayed for substrate range and select members from other phosphatase families (Q819K1, 1K7H, dog1 and dog2). The outer ring and legend indicate the position and colour scheme assigned to different phyla. The inner ring indicate those proteins that display activity on G6P (green squares), those with no activity on G6P (brown squares) and the phosphatase in the pathway under investigation (red square). The data is based on the publication by Huang et al., 2015 [1].

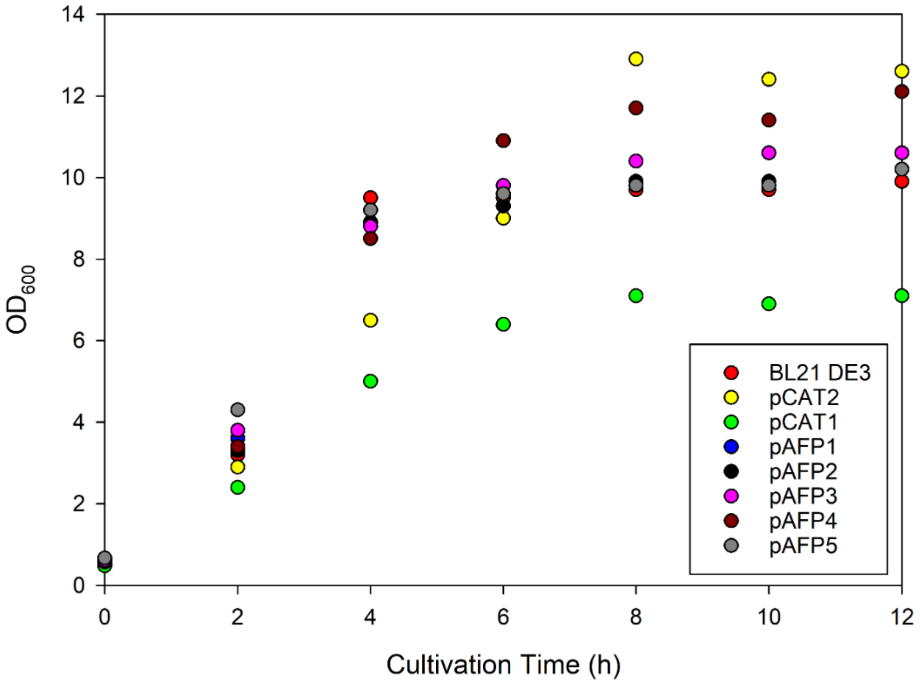

Figure S8. Growth curve of shake flask culture of various recombinant *E. coli* constructs containing different permutated ORFs with *E. coli* BL21 DE3 empty vector as control strain. IPTG inductions were done at $t = 1$ h. All plots represent the mean values obtained from duplicate experiments.

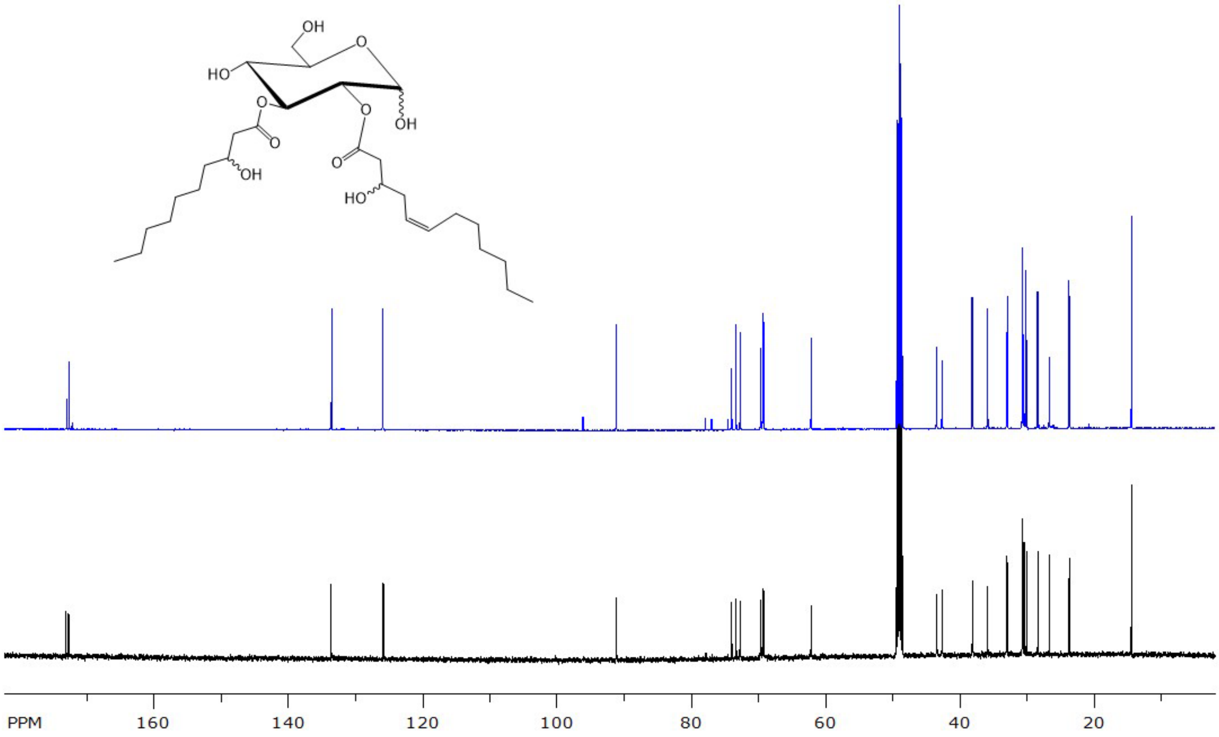

Figure S9. Comparison of ^{13}C NMR spectra of the *E. coli* pCAT2's glucosedilipid (black) and glucosedilipid from *R. badensis* DSM 100043^{T} (blue) in methanol-*d4* at 150 MHz confirming identical structure. Additional small NMR signals in the upper arise from the α/β anomeric equilibrium in solution.

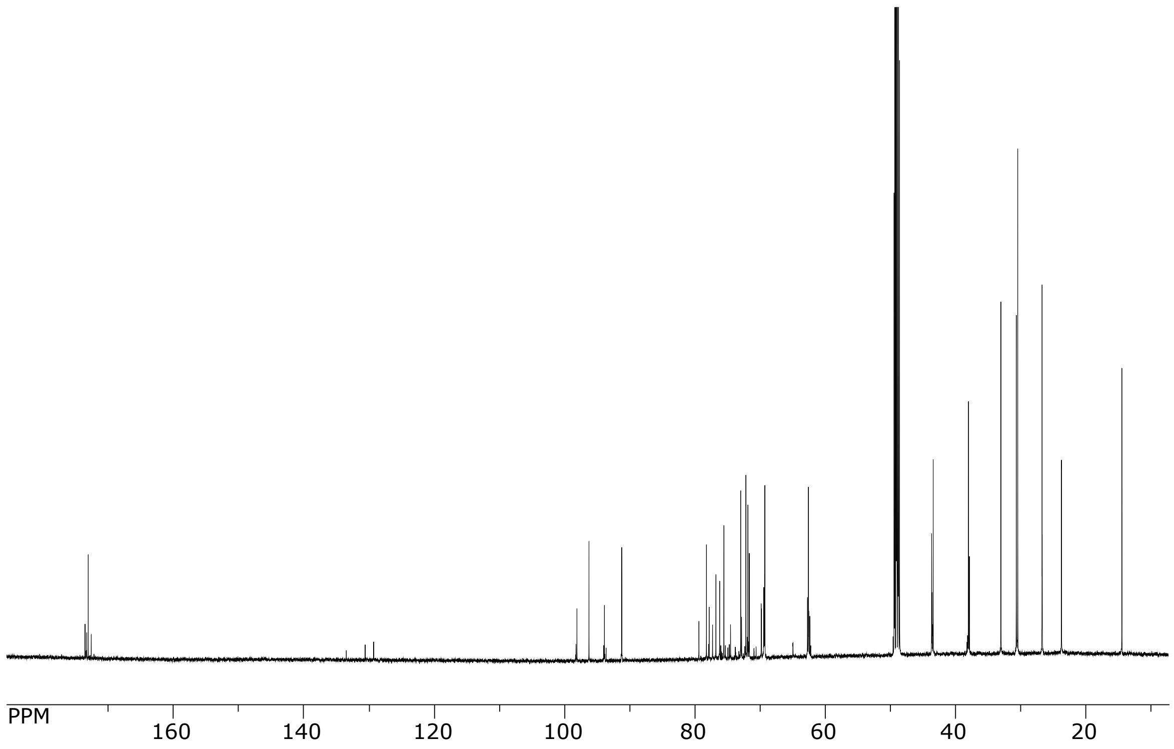

Figure S10. ^{13}C NMR spectrum of glucosemonolipid Glu-$C_{10:0}$ (fraction 37) from *E. coli* pAFP1 in methanol-d_4 at 150 MHz.

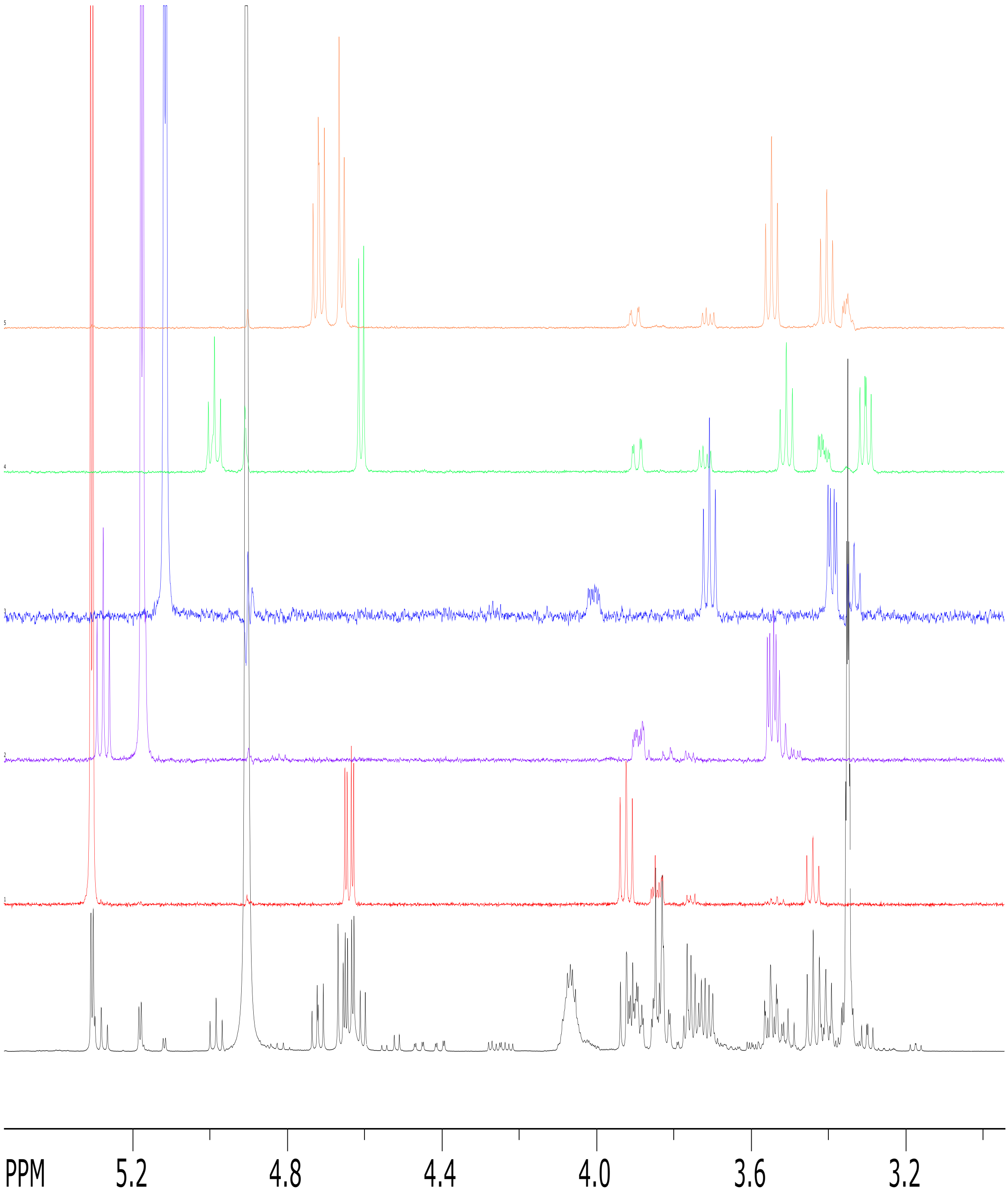

Figure S11. Expansion of ^{1}H NMR spectrum of glucosemonolipid Glu-$C_{10:0}$ (fraction 37) from *E. coli* pAFP1 and selective 1D TOCSY spectra displaying the individual glucopyranosyl ^{1}H spinsystems of the glucose monolipids **1**–**3** identified in fraction 37 in methanol-d_4 at 600 MHz. Trace 1: α-anomer of **1**, trace 2: α-anomer of **2,** trace 3: α-anomer of **3**, trace 4: β-anomer of **2**, trace 5: β-anomer of **1**.

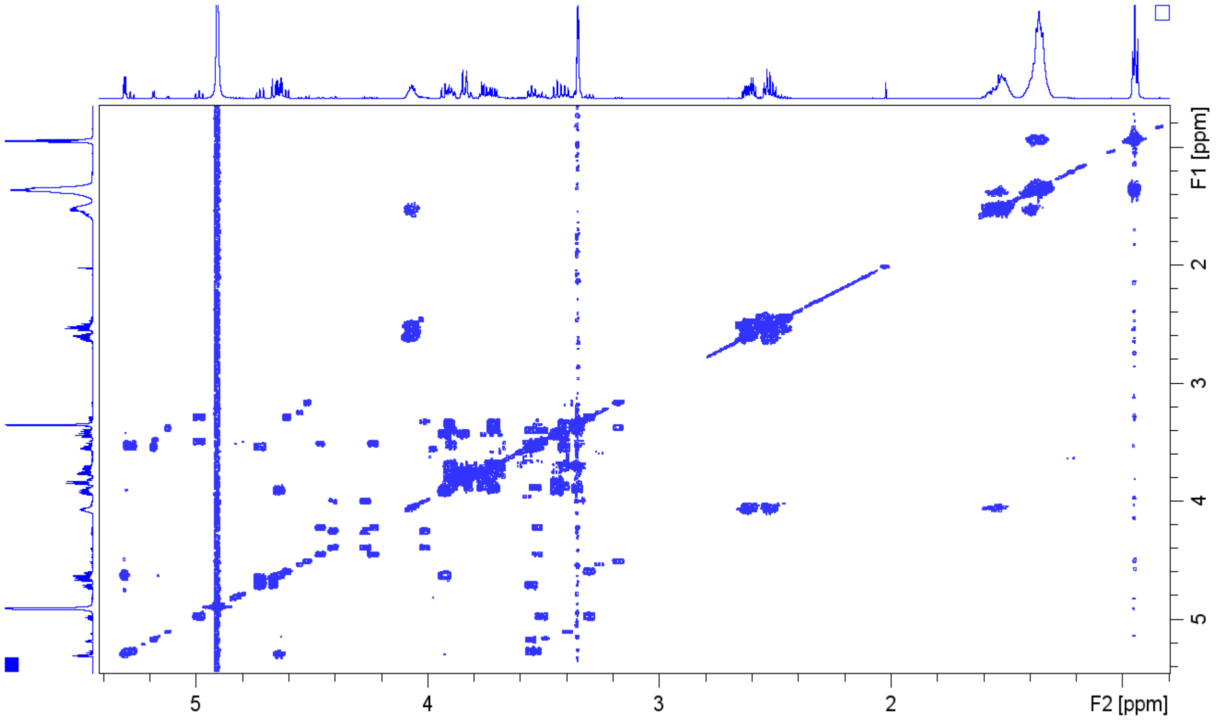

Figure S12. 2D COSY spectrum of glucosemonolipid Glu-$C_{10:0}$ (fraction 37) from *E. coli* pAFP1 in methanol-d_4 at 600 MHz.

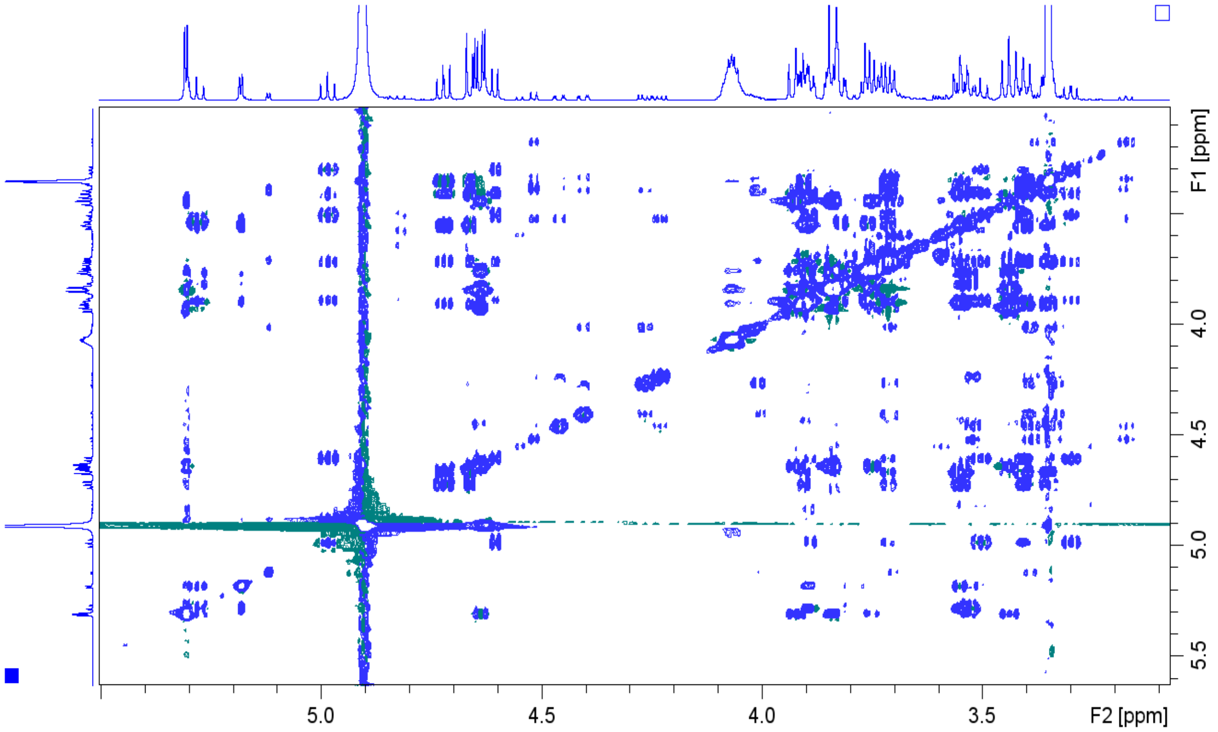

Figure S13. Expansion of 2D TOCSY spectrum of glucosemonolipid Glu-$C_{10:0}$ (fraction 37) from *E. coli* pAFP1 displaying the glucopyranosyl ^{1}H spinsystems of the glucose monolipids **1–3** identified in fraction 37 in methanol-d_4 at 600 MHz.

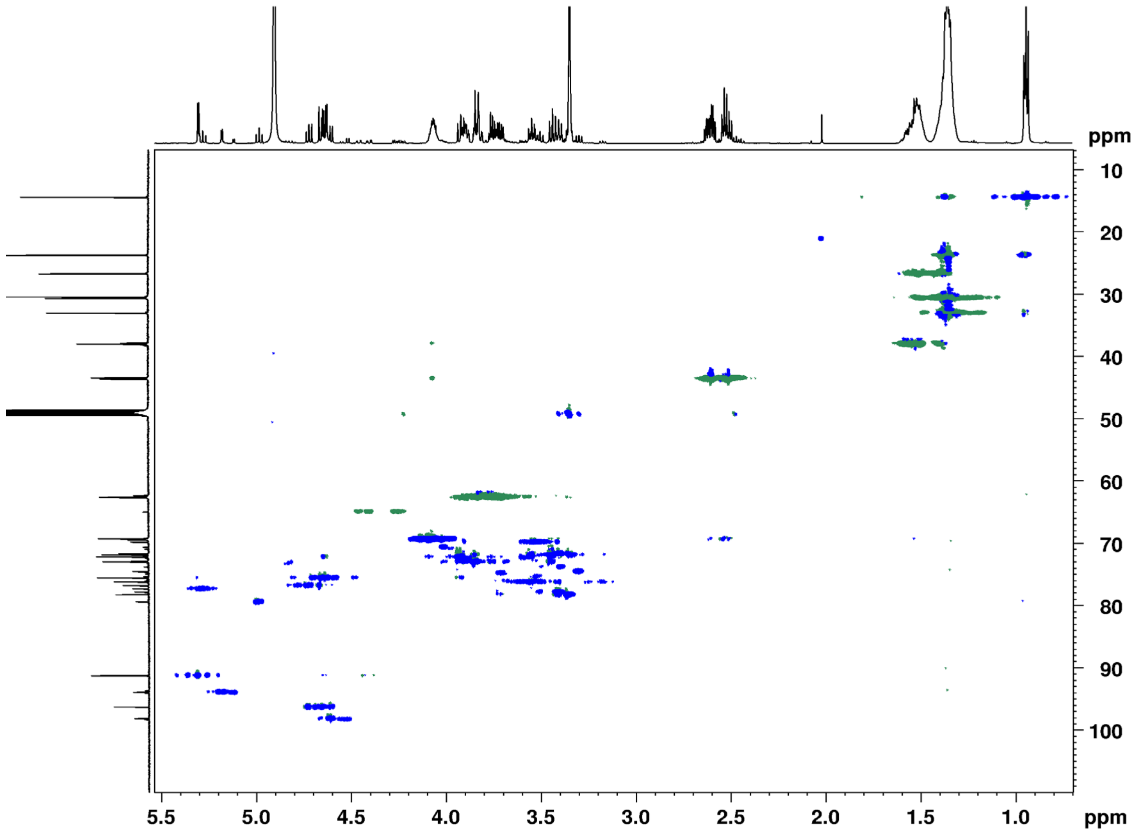

Figure S14. 2D gHSQC spectrum of glucosemonolipid Glu-$C_{10:0}$ (fraction 37) from *E. coli* pAFP1 in methanol-d_4 at 600 MHz.

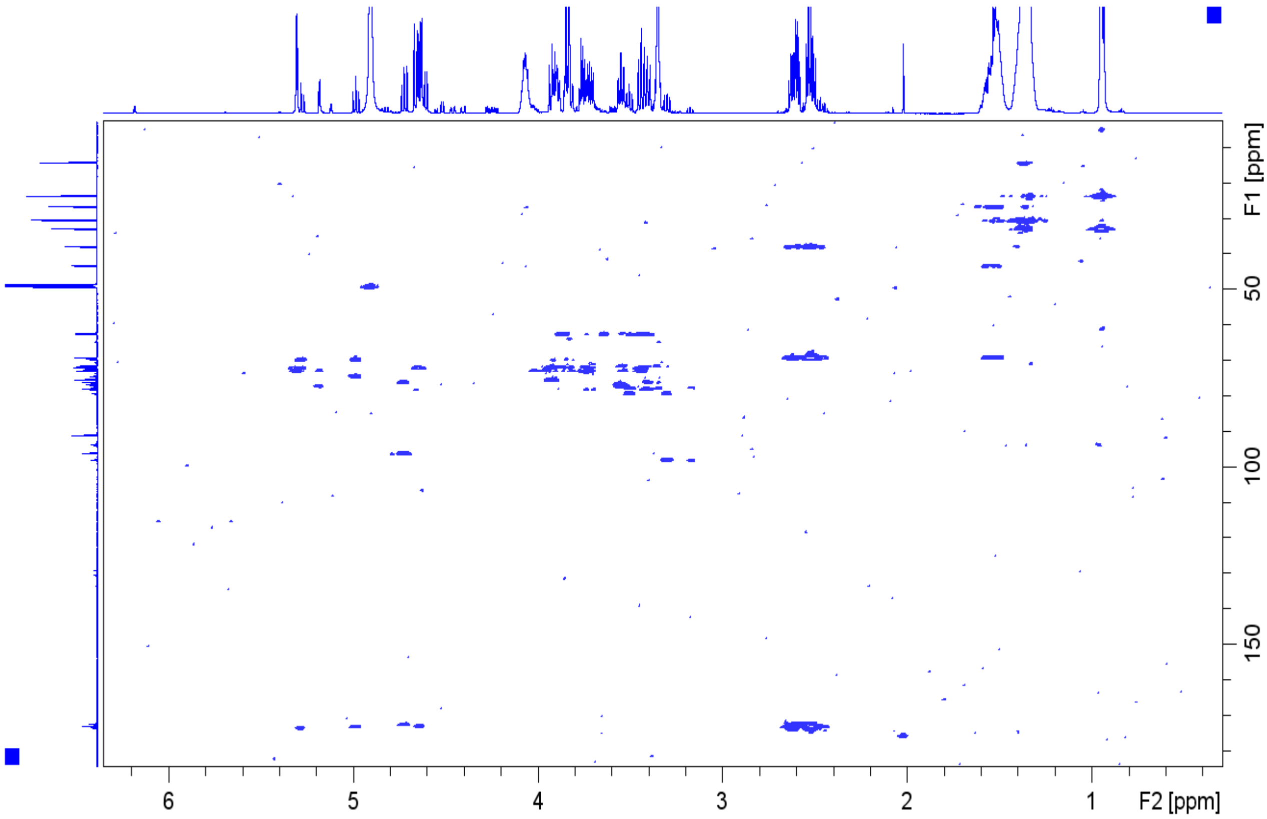

Figure S15. 2D gHMBC spectrum of glucosemonolipid Glu-$C_{10:0}$ (fraction 37) from *E. coli* pAFP1 in methanol-d_4 at 600 MHz.

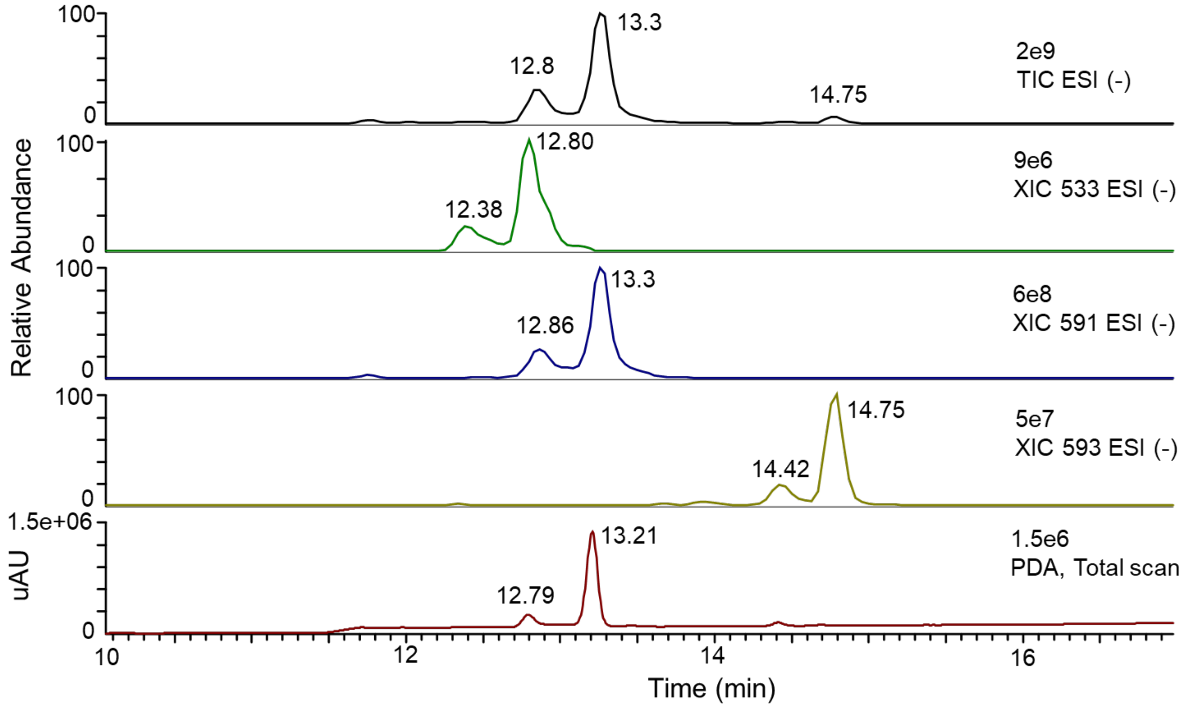

Figure S16. LC-ESI-MS/MS analysis in negative ion mode of the purified glucosedilipids produced by *E. coli* pCAT2 (pooled fraction 46-51). From top to bottom: total ion chromatogram (TIC) showing overall glucosedilipid congeners; extracted ion chromatogram (XIC) of Glu-$C_{10:0}$-$C_{11:0}$ isomers at RT 12.38 and 12.80 min; XIC of Glu-$C_{10:0}$-$C_{12:1}$ isomers at RT 12.86 and 13.3 min; XIC of Glu-$C_{10:0}$-$C_{12:0}$ at RT 14.42 and 14.75 min; and photodiode array (PDA) total scan between 190 and 400 nm.

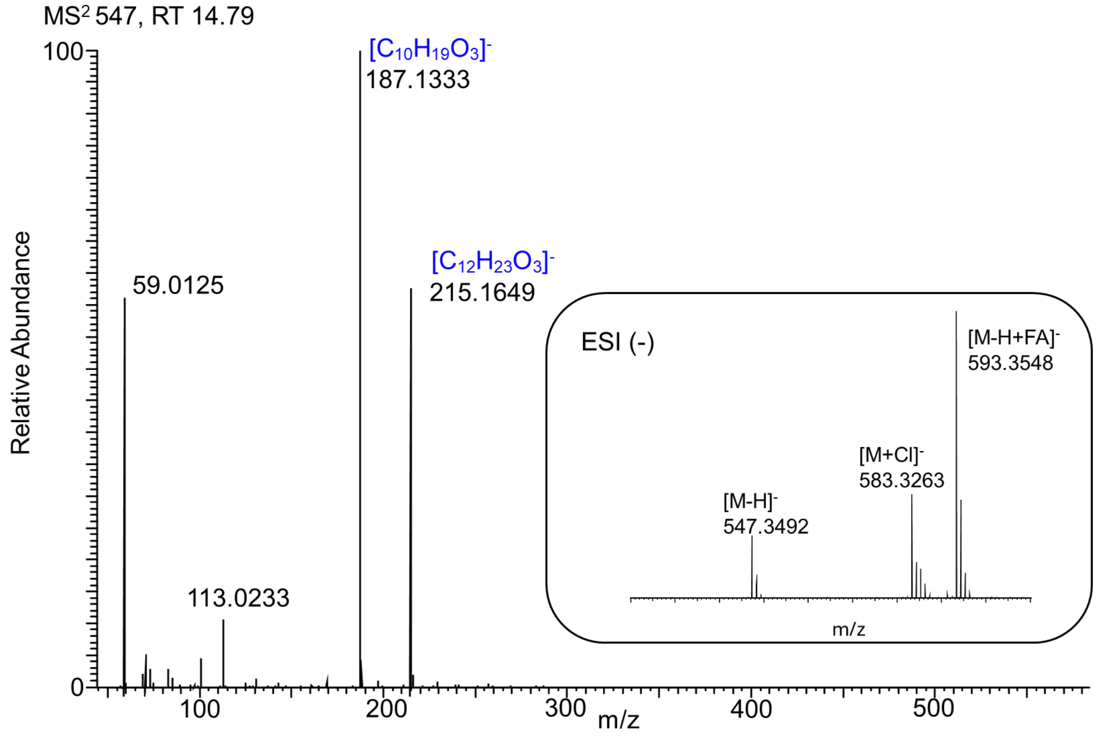

Figure S17. Mass spectra (MS^2 and ESI full MS (inset)) glucosedilipid Glu-$C_{10:0}$-$C_{12:0}$ as one minor congener produced by *E. coli* pCAT2.

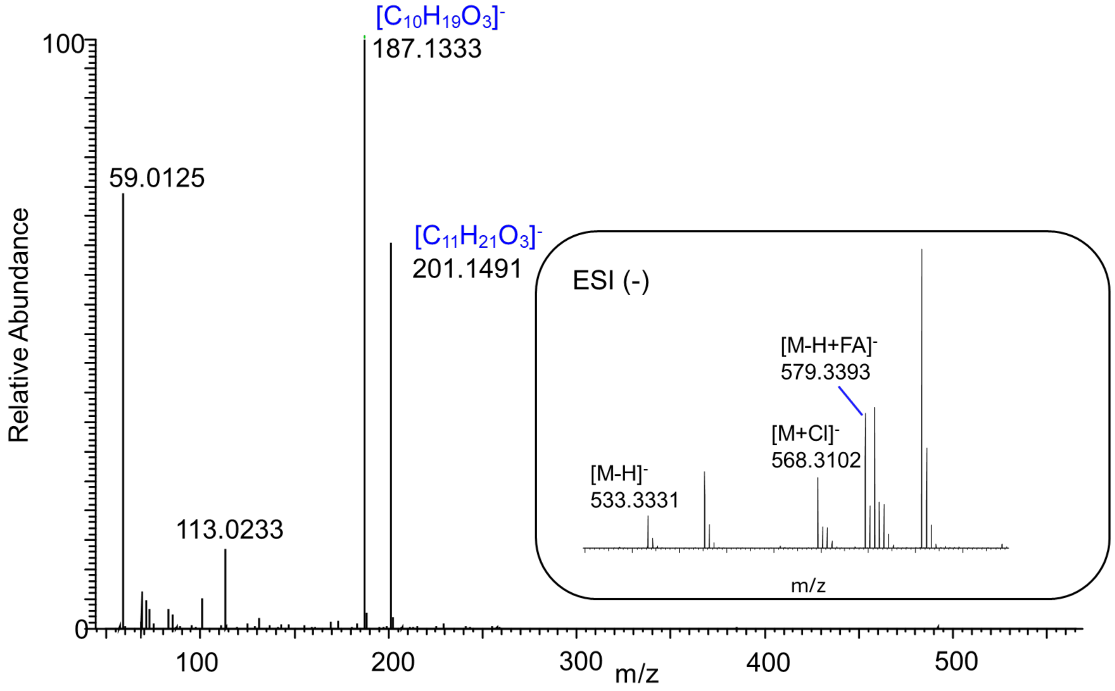

Figure S18. Mass spectra (MS[2] and ESI full MS (inset)) glucosedilipid Glu-$C_{10:0}$-$C_{11:0}$ as one minor congener produced by *E. coli* pCAT2.

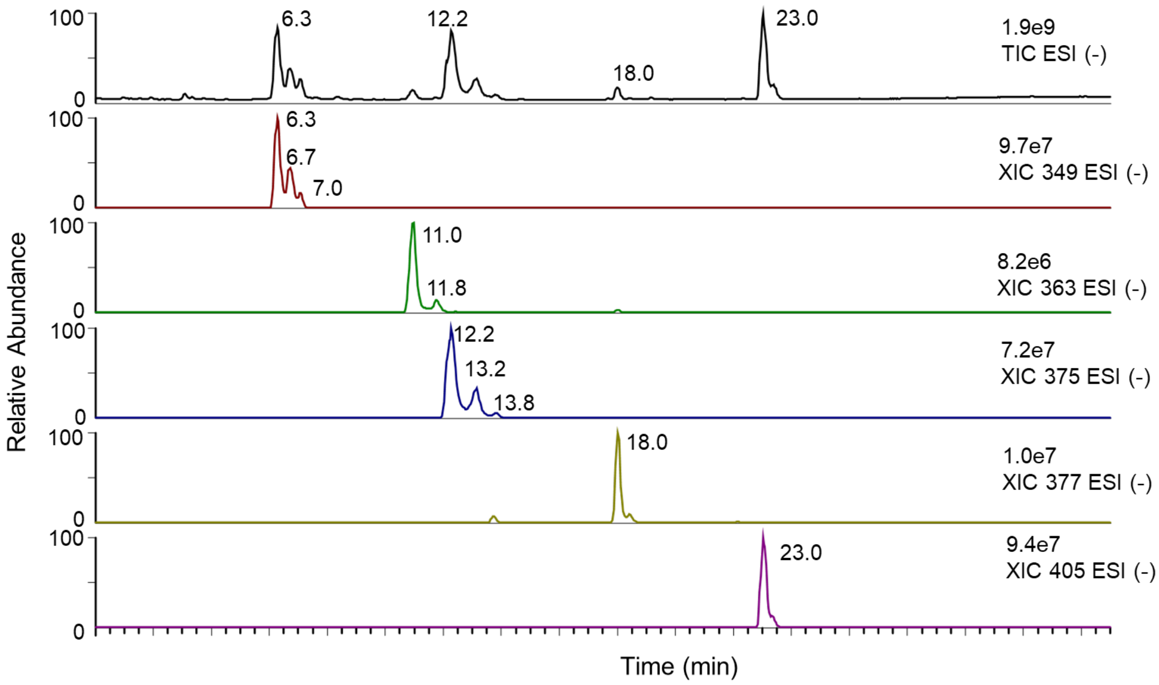

Figure S19. LC-ESI-MS/MS analysis in negative ion mode of the purified glucosemonolipids produced by *E. coli* pAFP1 (pooled fraction 37-40) From top to bottom: total ion chromatogram (TIC) showing overall glucosemonolipid congeners; extracted ion chromatogram (XIC) of Glu-$C_{10:0}$ isomers with *m/z* $[M-H]^-$ of 349.186; XIC of Glu-$C_{11:0}$ isomers with *m/z* $[M-H]^-$ of 363.202; XIC of Glu-$C_{12:1}$ with *m/z* $[M-H]^-$ of 375.202; XIC of Glu-$C_{12:0}$ with *m/z* $[M-H]^-$ of 377.218; and XIC of Glu-$C_{14:0}$ with *m/z* $[M-H]^-$ of 405.249.

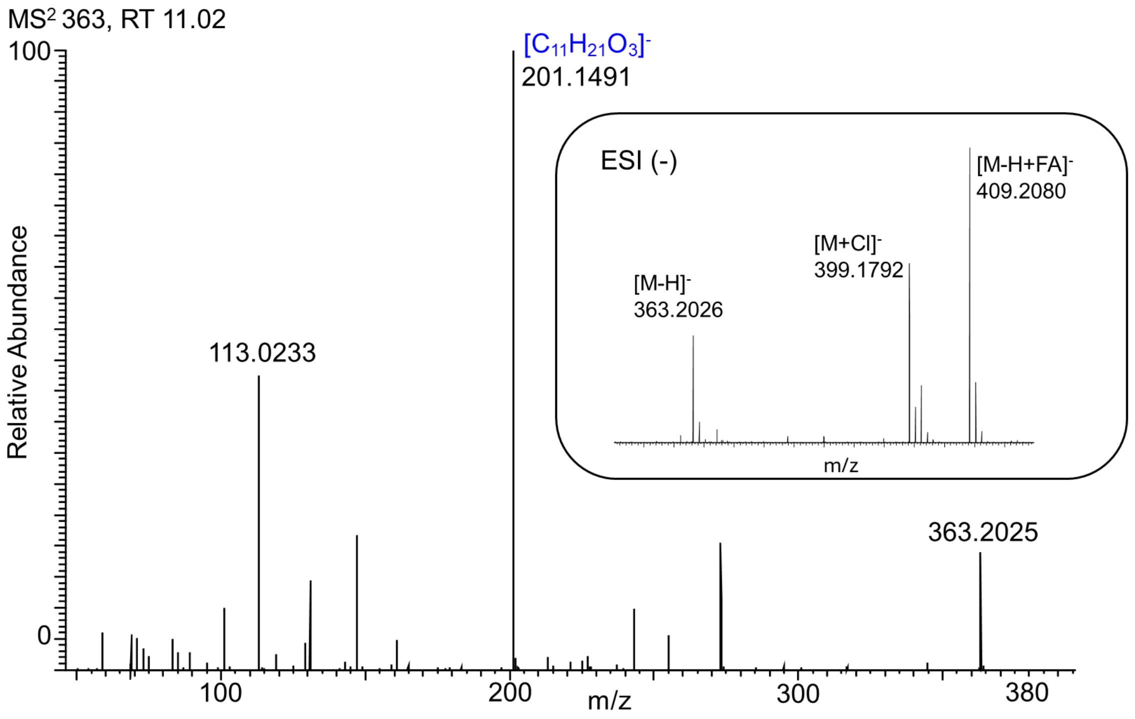

Figure S20. Mass spectra (MS^2 and ESI full MS (inset)) glucosemonolipid Glu-$C_{11:0}$ as one minor congener produced by *E. coli* pAFP1.

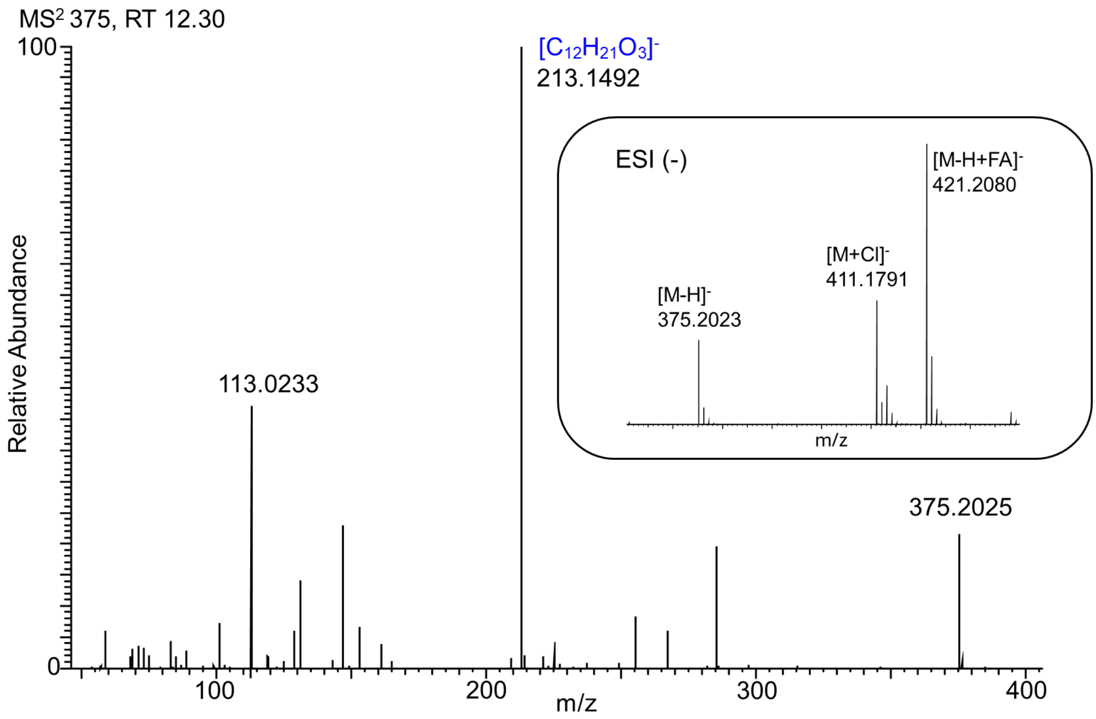

Figure S21. Mass spectra (MS^2 and ESI full MS (inset)) glucosemonolipid Glu-$C_{12:1}$ as one minor congener produced by *E. coli* pAFP1.

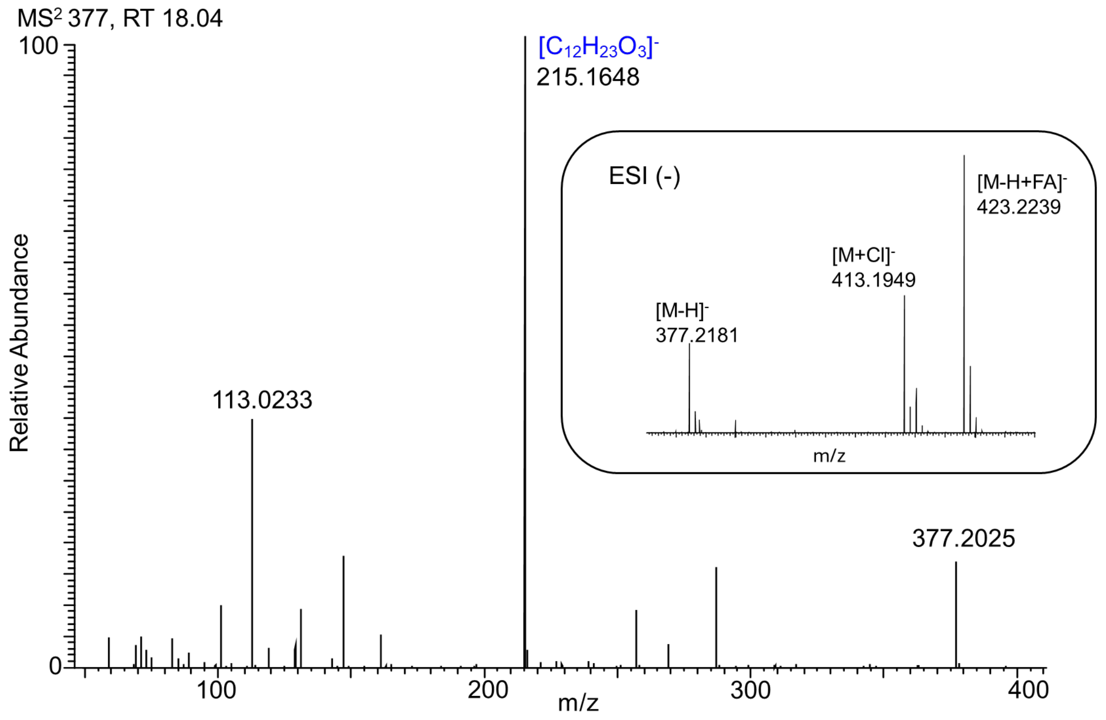

Figure S22. Mass spectra (MS² and ESI full MS (inset)) glucosemonolipid Glu-$C_{12:0}$ as one minor congener produced by *E. coli* pAFP1.

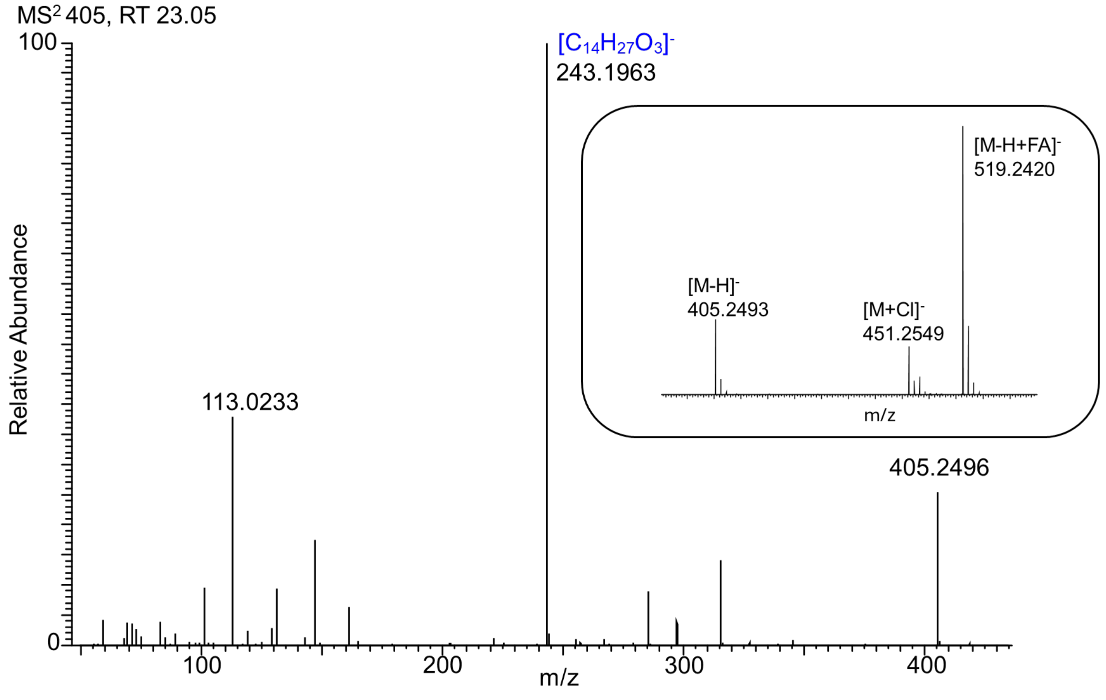

Figure S23. Mass spectra (MS² and ESI full MS (inset)) glucosemonolipid Glu-$C_{14:0}$ as one minor congener produced by *E. coli* pAFP1.

References

1. H. Huang *et al.*, “Panoramic view of a superfamily of phosphatases through substrate profiling,” *Proc Natl Acad Sci U S A*, vol. 112, no. 16, 2015, doi: 10.1073/pnas.1423570112.
